# Microscale Obstacle Resolving Air Quality Model Evaluation with the Michelstadt Case

**DOI:** 10.1155/2013/781748

**Published:** 2013-08-21

**Authors:** Anikó Rakai, Gergely Kristóf

**Affiliations:** Department of Fluid Mechanics, Budapest University of Technology and Economics, Budapest 1111, Hungary

## Abstract

Modelling pollutant dispersion in cities is challenging for air quality models as the urban obstacles have an important effect on the flow field and thus the dispersion. Computational Fluid Dynamics (CFD) models with an additional scalar dispersion transport equation are a possible way to resolve the flowfield in the urban canopy and model dispersion taking into consideration the effect of the buildings explicitly. These models need detailed evaluation with the method of verification and validation to gain confidence in their reliability and use them as a regulatory purpose tool in complex urban geometries. This paper shows the performance of an open source general purpose CFD code, OpenFOAM for a complex urban geometry, Michelstadt, which has both flow field and dispersion measurement data. Continuous release dispersion results are discussed to show the strengths and weaknesses of the modelling approach, focusing on the value of the turbulent Schmidt number, which was found to give best statistical metric results with a value of 0.7.

## 1. Introduction

Prognostic microscale obstacle resolving meteorological models and Computational Wind Engineering (CWE) models deal with the common fields of wind and pollutant dispersion modelling inside the urban canopy. Baklanov and Nuterman [1] show that these models with increasing computational capacity can be the final scale in a nested multiscale meteorological and dispersion model. In this paper the intersection of the two described disciplines is investigated with the assumptions of quasistationary flow and neutral meteorological conditions and the pollutant is considered a passive scalar.

Stull [2] defines microscale in meteorology as a few kilometers or less where the typical phenomena include mechanical turbulence caused by the buildings. Britter and Hanna [3] suggest the following lengthscales: regional (up to 100 or 200 km), city scale (up to 10 or 20 km), neighborhood scale (up to 1 or 2 km), and street scale (less than 100 to 200 m). The last two correspond to the microscale definition of Stull and are used here.

The general approach in CWE for air pollution dispersion modelling is adding a passive scalar transport equation decoupled from the solution of the flowfield or modelling Lagrangian particle dispersion in the computed flowfield. Although this is essentially less effort than computing the flowfield itself, if there are errors already in the mean velocity field, for example, reattachment length overestimation, or in the turbulent fields, for example, stagnation point anomaly, those errors are propagated in the pollutant transport modelling. Especially the turbulent fields which are not always vital for the flowfield are equally important in the dispersion model as they are responsible for the turbulent diffusion.

A comparison of the Eularian additional transport equation approach and the Lagrangian approach in a single building configuration is given in Gorlé et al.'s [4]. They find no great difference between the two approaches, but the additional transport equation has slightly better statistical results. However, the shape of the plume is significantly different between simulations and experiments. It must be added that the test case they are using has a pollutant source in the wake of the building, where the flow field modelling is already problematic, and they focus on the different modelling approaches of the flow field. Gorlé et al. [5] have investigated the effect of turbulent kinetic energy inlet boundary conditions on the dispersion results and compared to analytical Gaussian solutions. The test case was a simple boundary layer case, while in urban environments the effect of the inlet turbulent kinetic energy is smaller due to its extensive production in the shear layers around buildings.

Tominaga and Stathopoulos [6] investigated the effect of turbulent Schmidt number Sc_*t*_ on the dispersion results. The default value of this coefficient in general CFD applications is 0.7. The test cases they used are a free jet, a plume in the boundary layer, and dispersion around a single building. They found that a smaller value of Sc_*t*_ = 0.3 provides better predicted results on concentration distribution around plumes in open country and around a single building, where the turbulent momentum diffusion is often underestimated when using Reynolds Averaged Navier Stokes (RANS) models.

Due to the analogy existing between transport of momentum, heat and scalar concentration, improvement methods similar to the ones for the momentum equations, and heat transfer model developments can also be applied. Anisotropic models were developed for passive scalar dispersion problems focusing on heat transfer applications and are getting more and more popular in turbomachinery; see [7]. It was only used in Yee et al. [8] and Izarra [9] for pollutant dispersion problems in atmospheric applications. Yee et al. [8] did not compare it to isotropic models while Izarra [9] found improvement in some cases but worse results in others, showing clear disadvantages of the model. Both of them used the approach for more simple geometries.

This paper shows the effects of these two modelling questions, the value of Sc_*t*_, and the anisotropic approach on the dispersion results for a complex urban geometry, Michelstadt. This is an idealized city based on Central European city centres like Hannover and Cologne. For this test case both detailed flow field and dispersion measurements are available in the framework of the *COST Action ES 1006 on the evaluation, improvement, and guidance for the use of local-scale emergency prediction and response tools for airborne hazards in built environments* [10]. The effect of the numerical discretization on the flow field was investigated in Rakai et al. [11]; here the dispersion results are evaluated with four different mesh types, a tetrahedral, a polyhedral, and a Cartesian hexahedral and a body fitted hexahedral mesh. At least three different resolutions are used so that numerical uncertainty estimation can also be carried out.

## 2. Methods

**Figure 1:**
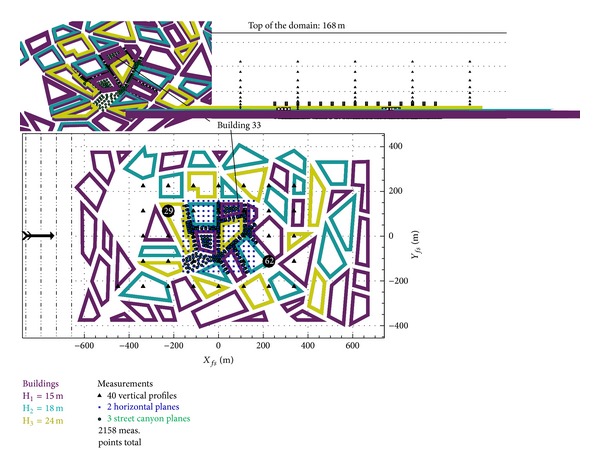
Computational domain with roughness elements, buildings, and flow measurement positions.

The used dataset, Michelstadt, is part of CEDVAL-LES, a collection of data for validation of Large Eddy Simulation (LES) models (http://www.mi.uni-hamburg.de/Data-Sets.6339.0.html). The geometry is an idealized Central-European city centre placed in the Atmospheric Boundary Layer (ABL) modelled by roughness elements. Two component velocity data time series were collected with Laser Doppler Velocimetry (LDV) in 40 vertical profiles, 2 horizontal planes, and 3 street canyon planes (see Figure 1). For the approach flow 3 component measurements were carried out.

**Figure 2:**
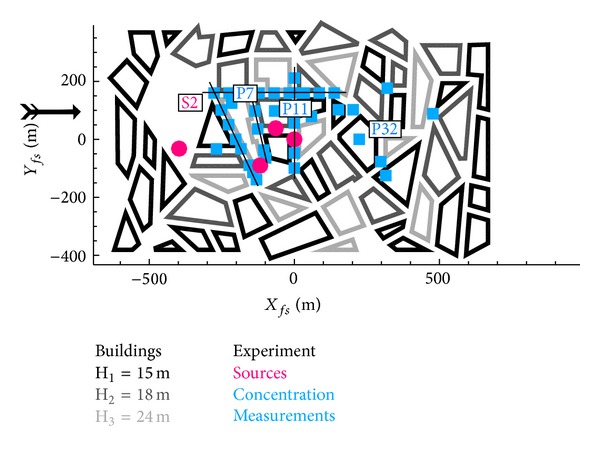
Michelstadt with dispersion measurement points for source S2, profile locations numbered P7, P11, and P32.

A detailed description of the flow measurements can be found in Hertwig et al. [12] and Berbekar [13] describes the dispersion measurements. For the dispersion different source locations were used, from which here only results for source S2 (see Figure 2) are calculated. 58 measurement locations were measured for this source which can also be seen in Figure 2. A constant plane at height *z* = 7.5 m was measured and at three locations vertical profiles are also available. The source is built in the wind tunnel's floor.

The computational domain was defined to correspond with the COST 732 Best Practice Guideline, Franke et al. [14], which resulted in a 1575 × 900 × 168 m^3^ domain, with a distance of the buildings of 11*H*_3_ from the inflow, 9.4*H*_3_ from the outflow and at least 6*H*_3_ from the top boundaries, where *H*_3_ = 24 m is the highest buildings' height (see Figure 1). The computations were done in full scale while the experiment was done at a scale of 1 : 225. The dependence of the results on this scale change was investigated by Franke et al. [15] using both full scale and wind tunnel scale simulations and no significant difference in the statistical validation metrics was observed for the flow field. As can also be seen in Figure 1, four lines of the wind tunnel's roughness elements were included in the mesh as the first buildings are in the wake of them. The heights of the roughness elements are 9 and 18 m in full scale, so they are relatively high compared to the buildings and the corresponding aerodynamic roughness height *z*_0_ = 1.53 m is unattainable with reasonable meshes.

**Figure 3:**
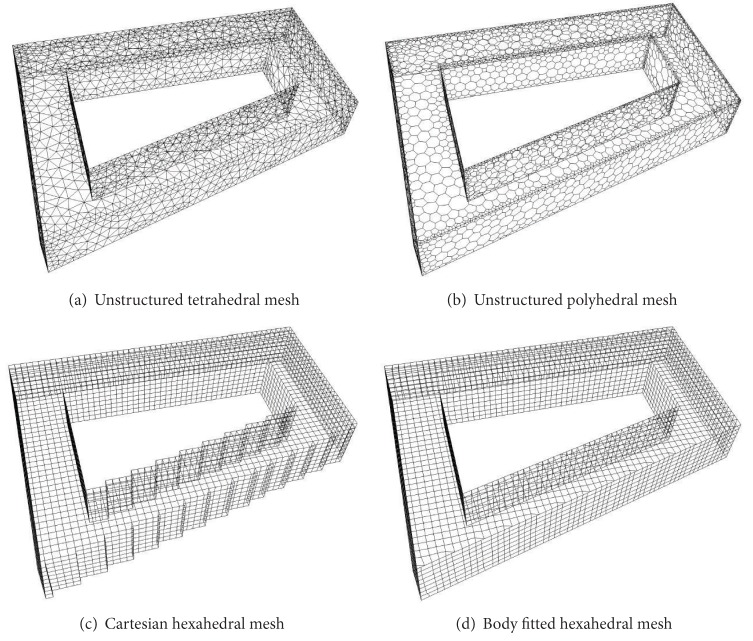
Coarsest surface meshes on Building 33; see Figure 1.

Four different mesh type results are investigated, a tetrahedral, a polyhedral, a Cartesian hexahedral, and a body fitted hybrid, mostly hexahedral. All of them are automatically generated which is important to enable us to carry out these kinds of simulations for any urban geometry. We used ANSYS Icem to generate the tetrahedral meshes and ANSYS Fluent [16] to convert them to the polyhedral meshes. For the hexahedral meshes, snappyHexMesh, the mesh generator of OpenFOAM was used. More details on the quality of the meshes and its generation can be found in Rakai et al.'s [11]; here only the typical surface meshes are shown in Figure 3 and the resulting cell numbers can be found in Table 1.

The Eulerian approach is considered for the calculation of passive scalar dispersion, using the results of the flowfield described in Rakai et al. [11]. The transport equation of the mean passive scalar concentration *c* can be seen in (1), with *u*_*j*_ mean velocity vector, *D* molecular diffusion, *Q* source, and $\bar{u_{j}^{\prime }c^{\prime }}$ turbulent scalar flux:

(1) $$\begin{array}{c} \partial_{t}c+\partial_{j}\left(u_{j}\cdot c\right)=\partial_{j}\left(D\cdot \partial_{j}c\right)-\partial_{j}\left(\bar{u_{j}^{\prime }c^{\prime }}\right)+Q. \end{array}$$

The closure of the turbulent scalar flux in general is

(2) $$\begin{array}{c} \bar{u_{j}^{\prime }c^{\prime }}=-D_{jk}\partial_{k}c. \end{array}$$

In most of the cases in CWE, *D*_*jk*_ is defined as a scalar field computed from the turbulent viscosity *ν*_*t*_ divided by the turbulent Schmidt number Sc_*t*_:

(3) $$\begin{array}{c} D_{jk}=\frac{\nu_{t}}{\text{S}{\text{c}}_{t}}. \end{array}$$

But it can also be defined as a tensor using an anisotropic approach, see Yee et al. [8], as in (4) with turbulent kinetic energy *k*, its dissipation *ɛ*, and the mean velocity gradient tensor *u*_*j*,*k*_:

(4) $$\begin{array}{c} D_{jk}=C_{s1}\frac{k^{2}}{ɛ}\delta_{jk}+C_{s2}\frac{k^{3}}{{ɛ}^{2}}\left(u_{j,k}+u_{k,j}\right). \end{array}$$

The constants are taken as

(5) $$\begin{array}{c} C_{s1}=0.134C_{s2}=-0.032. \end{array}$$

For the comparison of experimental and simulation results a dimensionless concentration, *c*_∗_, is defined in (6), with the reference velocity *U*_ref_, a reference length *L*, and the source strength *Q*_source_:

(6) $$\begin{array}{c} c_{*}=\frac{c\cdot U_{\text{ref}}\cdot L^{2}}{Q_{\text{source}}}. \end{array}$$

For the comparison three different statistical metrics are used. Using matrix norms for comparison is the simplest approach; here the *L*2 norm is used; see (7). This metric can be seen as a normalized relative error of the whole investigated dataset:

(7) $$\begin{array}{c} L2=\frac{\sqrt{\sum_{i=1}^{n}{\left(E_{i}-S_{i}\right)}^{2}}}{\sqrt{\sum_{i=1}^{n}E_{i}^{2}}}. \end{array}$$

*E*_*i*_ and *S*_*i*_ are the corresponding experimental data and simulation results in the *i*th experimental point, with a total of *n* experimental points.

The factor of two (FAC2, see (8)) metrics often used in air quality model evaluations, see Chang and Hanna [17], is also used to avoid judgment by only one, probably biased metric:

(8) $$\begin{array}{c} \text{FAC}2=\frac{N}{n}=\frac{1}{n}\sum_{i=1}^{n}N_{i}\;\text{with}\\ N_{i}=\{\begin{array}{cc} 1, & \text{for}\;0.5\leq \frac{S_{i}}{E_{i}}\leq 2.0\\ 0, & \text{for}\;\text{else}. \end{array} \end{array}$$

As in air quality modelling the results may differ in several orders of magnitude, an additional metric from Chang and Hanna [17] is used which is more sensitive to the changes in the order of the results, so the small differences are not hidden by the large order values. The metric chosen is MG, geometric mean bias:

(9) $$\begin{array}{c} \text{MG}=\exp\left(\bar{\ln E_{i}}-\bar{\ln S_{i}}\right). \end{array}$$

The boundary conditions, model and numerical setting for the flow field calculations were described in Rakai et al. [11]. For the dispersion calculations zero gradient conditions were defined at all boundaries except for the inflow, where 0 value was given. The source was modelled as a constant volume source defined as the cell closest to the real source centre in the wind tunnel measurements. Calculations were carried out in a transient solver of OpenFOAM, scalarTransportFoam, modified to contain all parts of (1), until a steady state was reached. Results of the upwind and limited linearUpwind schemes for the convective term discretization, see [18], are both evaluated. The 1.7.1 version of OpenFOAM was used.

## 3. Results

In this section the results of the investigations are shown objectively, and evaluation and interpretation will be given in Section 4 with comparison to the relevant literature data.

### 3.1. Metric Evaluation

**Figure 4:**
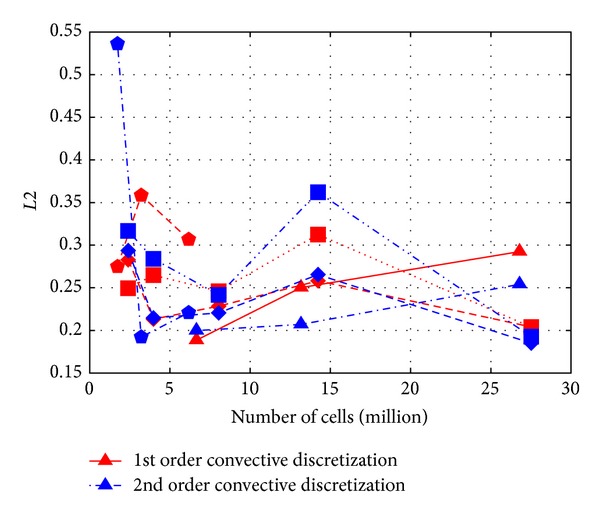
Values of *L*2 for *c*_∗_ as a function of cell number (triangle—tetrahedral, pentagon—polyhedral, square—Cartesian hexahedral, rotated square—body fitted hexahedral).

**Figure 5:**
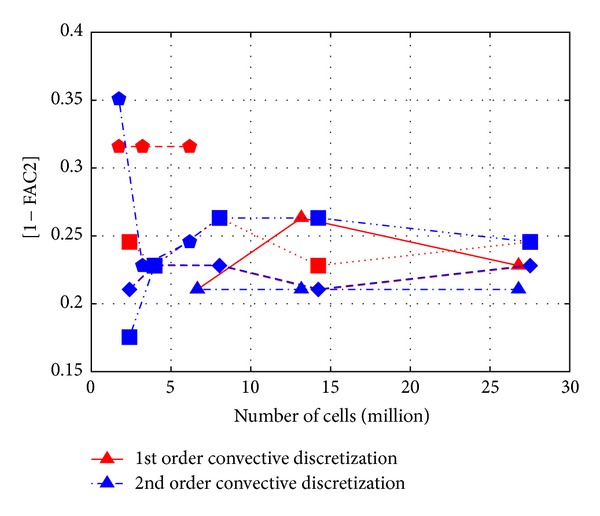
Values of 1 − FAC2 for *c*_∗_ as a function of cell number (triangle—tetrahedral, pentagon—polyhedral, square—Cartesian hexahedral, rotated square—body fitted hexahedral).

**Figure 6:**
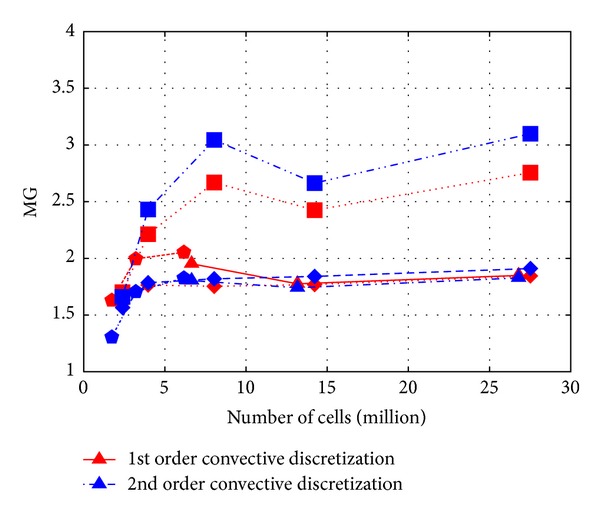
Values of MG for *c*_∗_ as a function of cell number (triangle—tetrahedral, pentagon—polyhedral, square—Cartesian hexahedral, rotated square—body fitted hexahedral).

At first it is interesting to look at the statistical metrics to check the overall performance of the transport model. *L*2 norm metric results are in Figure 4, 1 − FAC2 results in Figure 5, and MG results in Figure 6 as a function of cell number. Note that 1 − FAC2 is used for easier visual comparison, so that smaller values are always the better.

### 3.2. Effects of the Flow Field

**Figure 7:**
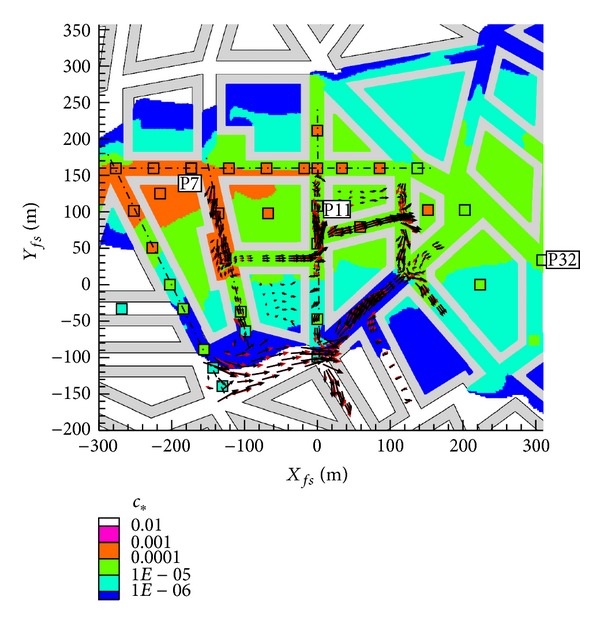
Possible effects of flow field on dispersion: 9 m flow vectors (black—experiment, red—simulation), 7.5 m dispersion results (square—experiment, contour—simulation) dash-dot line and numbers : location of profiles).

The dispersion measurements were carried out at a height of 7.5 m in full scale while flow field measurements were done only at 2 and 9 m. The results of the last are compared here together with the dispersion results in Figure 7. Flowfield results of the finest body fitted hexahedral mesh were used for the figure. Flowfield results were compared in more detail in Rakai and Franke [19] and Rakai [20]. What is important to recall here is that in the 2 m canyons there are generally larger differences between simulation and experiment results of the flow field, but those are further away from the dispersion measurement points at 7.5 m investigated here in detail.

### 3.3. Effects of Discretization

**Figure 8:**
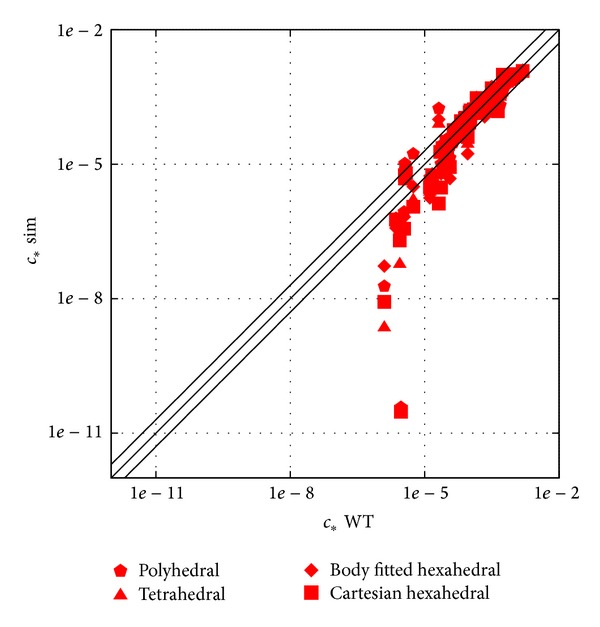
Scatter plot of the results from different mesh type simulations; results shown here are from the coarsest meshes, upwind convective discretization.

**Figure 9:**
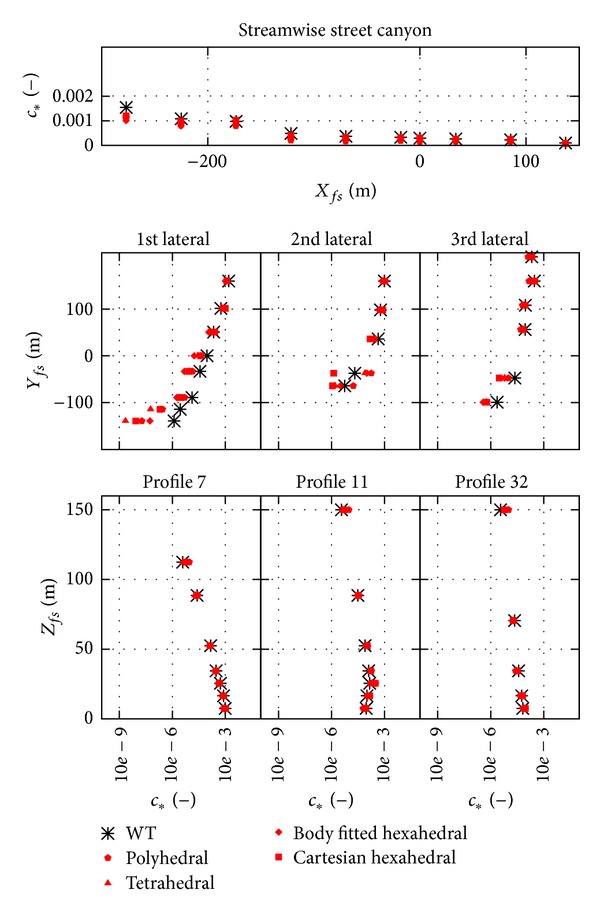
Profiles of the results from different mesh type simulations; results shown here are from the coarsest meshes, upwind convective discretization. Note that *c*_∗_ scales are logarithmic except for the streamwise profile.

The results of the four different mesh types are shown in Figure 8 as scatter plot and Figure 9 as profiles. The location of the profiles was shown in Figures 2 and 7. The results of the different mesh types show similar trends, and an underestimation of the spread of the plume can be observed for all different types in the first lateral street canyon both in Figures 8 and 9.

### 3.4. Sc_*t*_ Number Dependency

As one of the most argued and important constants of the conceptual model in dispersion calculations is the turbulent Schmidt number, Sc_*t*_, as discussed already in (1), the effect of its value on the dispersion results was investigated. Calculations were carried out varying its value from 0.1 to 1 with a resolution of 0.1 using second order discretization to reduce numerical diffusion which may change the picture.

**Figure 10:**
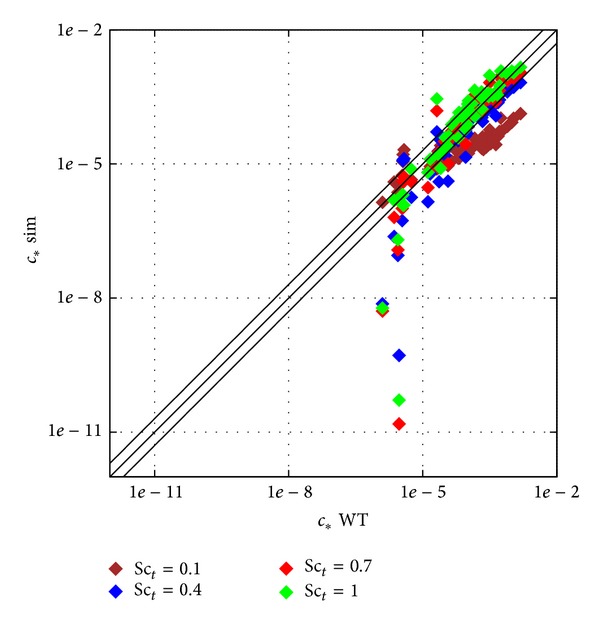
Scatter plot of the Sc_*t*_ dependency test on the coarsest body fitted hexahedral mesh.

**Figure 11:**
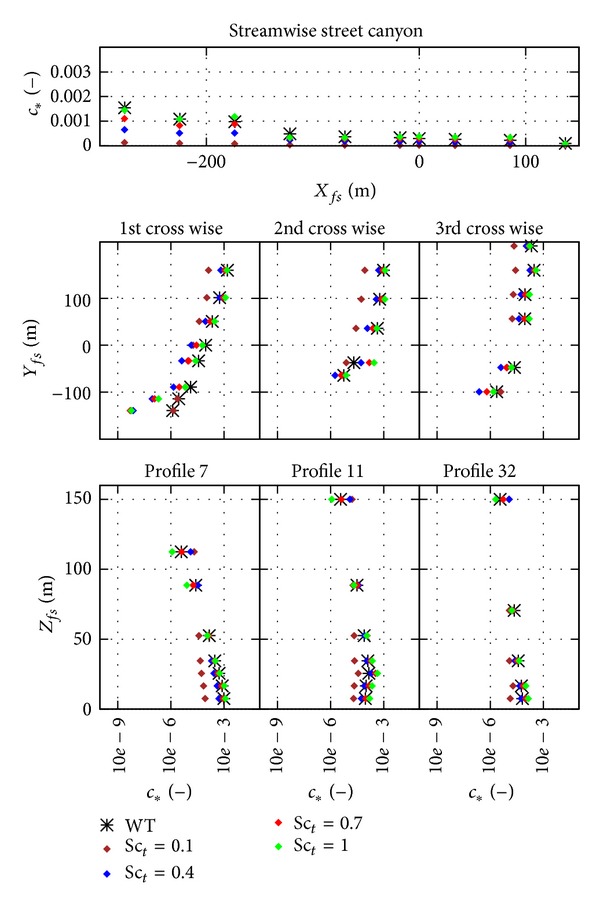
Profiles of the Sc_*t*_ dependency test on the coarsest body fitted hexahedral mesh.

The scatter plot and profiles already familiar from the previous comparison are shown in Figures 10 and 11 to see clearly the effect of the closure constant Sc_*t*_ on the results.

Putting a small value like Sc_*t*_ = 0.1 enhances turbulent diffusion very much (see (3)) so the crosswise profiles become more flat and pollution is spread out of the main streamwise street canyon. On the contrary, the bigger value like Sc_*t*_ = 1 reduces diffusion and more pollution remains in the streamwise street canyon. It is obvious from Figure 11 that in some locations one effect increases matching with the experiments while in other locations the opposite is observed.

### 3.5. Anisotropic Model Tests

It is worthwhile to investigate a different model closure, namely, an anisotropic model. As the recent investigations mentioned in Section 1 were carried out for simple geometries and no clear decision was made about their advantage over the isotropic model, it was decided to check their effect on the concentration field in this complex geometry. The first drawback of this more complicated model is that with the default settings used for the dispersion calculations convergence could only be obtained for the coarsest hexahedral meshes.

**Figure 12:**
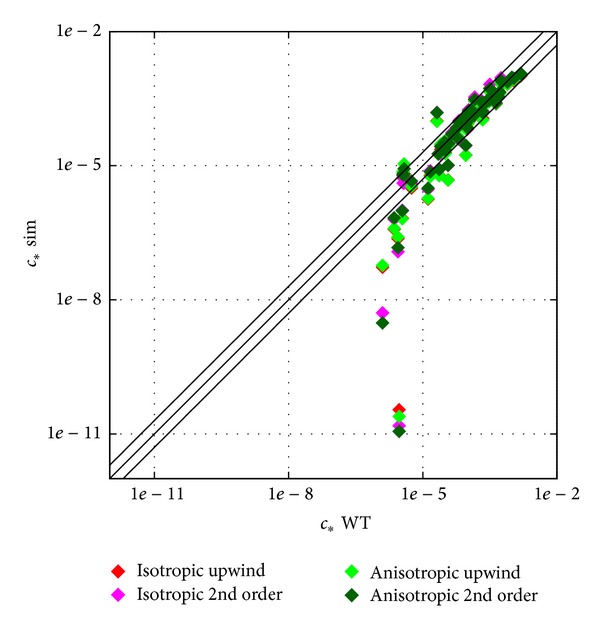
Scatter plot of the anisotropic model test, coarsest body fitted hexahedral mesh results.

**Figure 13:**
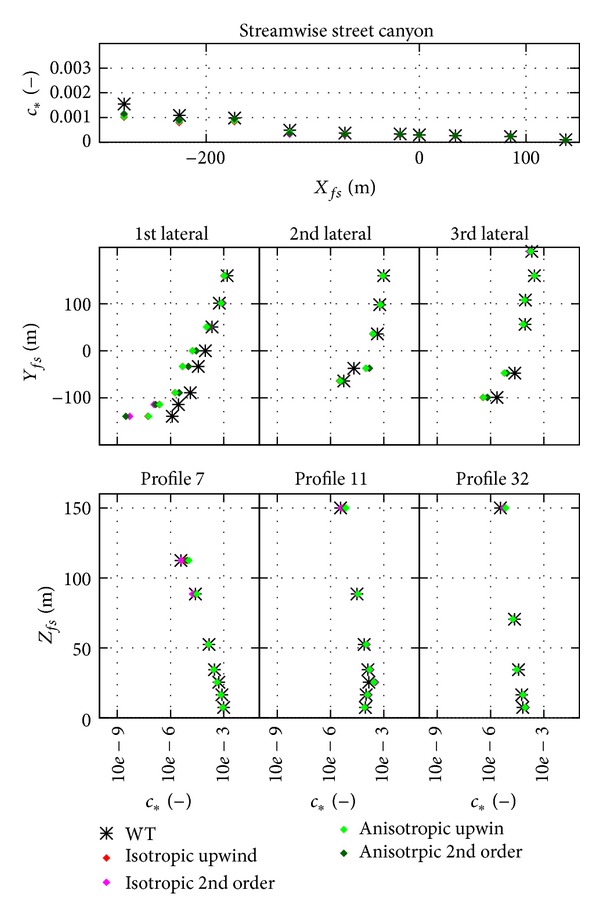
Profiles of the anisotropic model test, coarsest body fitted hexahedral mesh results.

This already would exclude the use of this model for operational purposes. However, the converged results are evaluated with the scatter plot and profile plots in Figures 12 and 13, respectively.

The *L*2 metric is given here for statistical comparison: 0.28 for the 1st order isotropic approach and 0.29 for the 2nd order, while for the anisotropic it is 0.24 and 0.23, respectively.

## 4. Discussion

In the following the results shown in Section 3 are discussed and interpreted according to the similar investigations in the literature.

### 4.1. Metric Evaluation

What is straightforward at first look from Figures 4, 5, and 6 is that all the expected properties, like improvement with mesh refinement and the second order differencing scheme, are not noticeable.

It is also important to point out that the choice of the metric has a considerable effect on interpreting the results. For example, the MG metric shows clearly poorer performance of the Cartesian hexahedral meshes for cases over 3 million cells, while this is not so apparent in case of the other metrics. This can be explained by the MG being a logarithmic metric taking more into consideration the underestimation of small values.

Looking at the metrics only, no explanation can be given on the behaviour of the mathematical models. In the following parts the concentration field will be investigated in more detail to find out the cause of the scattering metrics. As a first possible explanation the single transport equation must be recalled from (1). It contains the mean velocity field which was found to be modelled quite well in Rakai et al. [11], but some important deficiencies were found in the urban canopy, exactly where there are the concentration measurement points. The diffusion is governed by the turbulence field which was shown to have rather big differences from the measured values already. And additionally the gradient diffusion hypothesis is used for the turbulent diffusion fluxes; see (3) which has a single and often questionable closure constant, the turbulent Schmidt number.

The identification of the modelling problems should therefore deal with all these three aspects keeping in mind at the same time the numerical errors causing numerical diffusion which will as well change the picture.

All these sources of errors may easily end up causing the more or less stochastic picture of the dispersion results.

### 4.2. Effects of the Flow Field

With the limited points where the flow and dispersion measurements are close to each other it cannot be justified that deficiencies are caused by the flowfield, see Figure 7. The most problematic dispersion points, which are in the first lateral street canyon, apparently underestimating the spread of the plume in lateral direction, have no flowfield measurement data.

**Figure 14:**
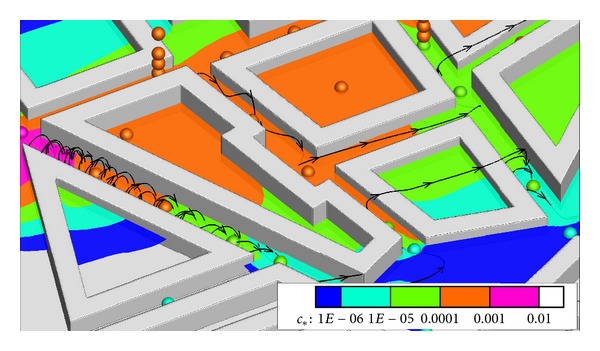
Streamlines in street canyons and 7.5 m dispersion results (sphere—experiment, contour—simulation).

To have a view on the flowfield in the streetcanyons, in Figure 14 three streamlines in the street canyons are shown together with the dispersion contour of simulations and points for experiments. As graphical visualization of all the cell values—what is necessary for the streamline calculation—is very demanding, the results of the coarsest body fitted hexahedral mesh were used for the figure.

The greatest underestimation of the spread of the plume by the simulation is in the street canyons where the intensive vortex circulation can be observed. This vortex in the simulation is rotating perpendicular to the length of the canyon, possibly blocking the lateral movement of the plume. This can cause a difference if in the experiments the vortex is less intensive. Unfortunately this guess cannot be verified without more experimental results.

Another important thing to remember here is that although the boundary conditions in the experiments are stationary in the averaged values, the flow is essentially time dependent, with vortex shedding around the bluff bodies which change the location of the vortices. Hertwig et al. [12] have shown that close to street intersections several locations have shown bimodal velocity distributions evaluated from the time series of the results.

As no further conclusion can be drawn from the effect of the flowfield, in the following the effects of the numerical discretization are investigated in more detail.

### 4.3. Effects of Discretization

From Figures 8 and 9 no significant difference can be observed between the different spatial discretization, that is, the different mesh types. Difference can only be observed at locations of large underestimation, where the tetrahedral meshes have the worst results.

What can be seen clearly in the scatter plot in Figure 8 is that all simulations are underestimating very low concentration values. This is not a general behaviour but rather a property of the experimental dataset. As a counterpart of this behaviour an overestimation can also be observed. The location of the overestimation is the top of the vertical profiles, see Figure 9, while underestimation happens at the edge of horizontal profiles and can be related back to the deficiency of the turbulent scalar flux closure not to model properly the dispersion of a Gaussian plum; see Gorlé et al. [5]. Although here the plume is not dispersed in the open atmospheric boundary layer but in the urban canopy, the effect looks the same.

**Figure 15:**
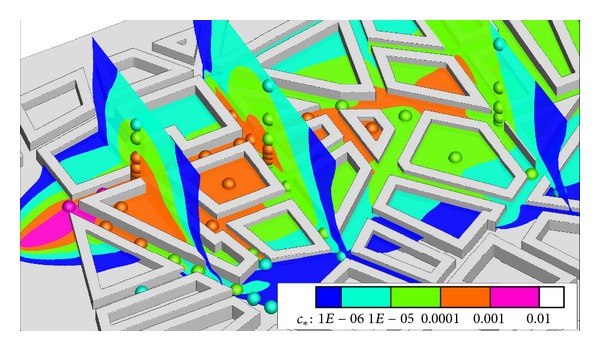
Cross-sections at profile locations with dispersion results (sphere—experiment, contour —simulation).

To maintain mass conservation this underestimation must be balanced, but probably no measurement points are located at those points where overestimation occurs. Also the *c*_∗_ values investigated differ several orders of magnitude, so this underestimation of small values can be balanced with small overestimation in higher *c*_∗_ regions. In Figure 15 this can be investigated with all the higher experimental results available, slices showing the shape of the plume.

At the slice closest to the source in Figure 15 the plume looks rather thin and seems to spread to a higher *z* value than in case of the measurement, the green contour containing the blue sphere. But this limited location is not enough to draw strong conclusions, and more measurement results would be necessary for these cross-sections. It is also important to remember that the scale is logarithmic, so to balance the underestimation in the edge of the plume for mass conservation, relatively weak overestimation is necessary.

The gradient diffusion hypothesis used here was criticized in Dezso-Weidinger et al. [21]. They carried out simultaneous measurements of the flow field and concentration field to evaluate the turbulent scalar fluxes, and in the urban canopy sometimes they had opposite direction of fluxes than the concentration gradient. Kukacka et al. [22] also carried out simultaneous flow and dispersion measurements, calculating convective and turbulent scalar fluxes in a street intersection geometry, which could be used to evaluate the model behaviour. In the Michelstadt experiment where flow field and dispersion measurements are not simultaneous and are not carried out at the same locations, no direct conclusions can be gained about the scalar fluxes. Later some model modifications will be shown to see if those improve the results.

With the refinement of the meshes the problem stated before does not diminish, and in most cases it is getting even worse. This was already concluded from the simple metric comparison in Figure 4. Profiles are very similar to the ones comparing the mesh type, so they are not shown here separately.

Before changing the conceptual model itself, it is important to estimate the numerical errors and uncertainties. The method described in the American Society of Mechanical Engineers (ASME) Standard for Verification and Validation in Computational Fluid Dynamics and Heat Transfer [23] was used combined with four different numerical uncertainty estimation methods of Phillips and Roy [24] to differentiate between discrepancies due to the coarse numerical resolution and the weaknesses of the conceptual model itself.

All methods estimate similar magnitude of numerical uncertainty; for more details see Rakai [20]. It was found that in the regions with the largest differences between experiments and simulation there are points which do not fall within the numerical uncertainty, so a possibility of conceptual model development is justified.

### 4.4. Sc_*t*_ Number Dependency

The default value in ANSYS Fluent is Sc_*t*_ = 0.7 (see [16]) but Tominaga and Stathopoulos [6] argue the value is defined for Fluid Mechanics test cases and not urban problems and Gorlé et al. [5] also show a lower optimal value for a test case of dispersion around a cube.

**Figure 16:**
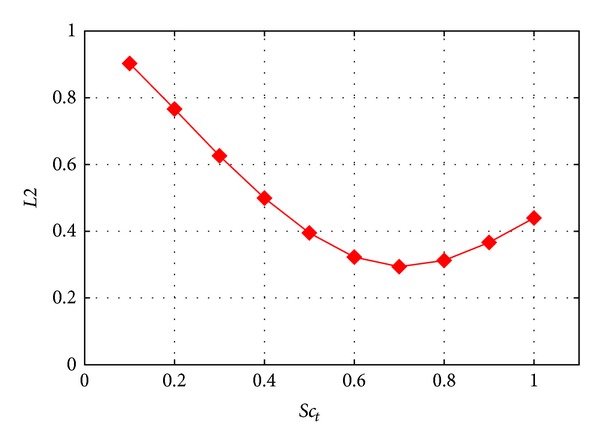
*L*2 metric as a function of Sc_*t*_.

In Figure 11 it was already shown that the change in Sc_*t*_ can improve in one location while worsening at another at the same time. For a quantitative measure to define an optimal value the already used *L*2 metric is shown in Figure 16 as a function of Sc_*t*_. The originally defined 0.7 value remains the best choice for this test case with the lower observed *L*2 metric.

One reason for this can be that Gorlé et al. [5] and Tominaga and Stathopoulos [6] focus on test cases around a single building, with detailed measurements in the wake of the building and the rooftop recirculation. The test case used here has a better represented measurement point distribution in the urban canopy, with buildings surrounding each other, which is a more realistic situation.

It can be concluded that for complex urban problems the Sc_*t*_ = 0.7 can still be regarded optimal, but for more specific geometrical problems other values can be valid. For operational modelling purposes where modelling of a complex urban canopy is the main goal, Sc_*t*_ = 0.7 is suggested.

### 4.5. Anisotropic Model Tests

It is difficult to evaluate the results from the plots in Figure 13; there is no clear trend what is changing for the anisotropic model. It is interesting to note, however, that both upwind and 2nd order convective discretization scheme results are shown, and the effect of numerical diffusion can be observed. For the 1st lateral profile, for example, upwind results are closer to the measured values, as the plume is spread more due to numerical diffusion. So the effect is similar to reducing Sc_*t*_ seen in Figure 11 and is stronger than the difference between the isotropic and anisotropic approach.

From statistical point of view this model has advantages as could be seen from the metric values in Section 3, but without having converged results for the finer meshes no more can be concluded. This can be understood if we have a look at Figure 4 with similarly low *L*2 values gained by mesh refinement as what was reached by using the anisotropic model.

This model clearly needs more investigation to justify its use and to increase its numerical stability. That would also be an opportunity to tune the model constants for urban applications which have not been done before.

## 5. Conclusions

A numerical experiment was carried out to model dispersion in a complex urban canopy geometry. Four different mesh types were investigated with at least three resolutions to enable solution verification with numerical uncertainty estimation. The weakest point in modelling the plume was identified as the edge of the plume in lateral direction where serious underestimation was found in the modelling results. This was clearly shown not to be a numerical error and model improvements were tested.

Changing Sc_*t*_ the dispersion model parameter could improve the plume edge values but with worse results at other locations. With detailed analysis of the dispersion pattern of a ground source it was found that the often questionable Sc_*t*_ value for this case is the optimal value of 0.7 as defined for academic Fluid Dynamics test cases.

Anisotropic modelling was also tested which was shown to have potential by comparing statistical metrics. Further model investigations are necessary to justify the results as convergence could be reached only with the coarsest mesh. Numerical stability must be increased and the model coefficients could also be optimized for urban flows.

To investigate in more detail the dispersion of the plume more detailed measurements would be favourable in vertical directions, combined with flow measurements at the same locations. As already mentioned simultaneous measurements for flow and dispersion were carried out by Dezso-Weidinger et al. [21] in a street canyon. For a street intersection Kukacka et al. [22] have carried out simultaneous measurements from which they computed vertical advective and turbulent scalar fluxes which could be a useful test case to investigate the dispersion model in more detail.

## Figures and Tables

**Table 1:** Cell numbers (million cells) of the investigated meshes.

	Coarsest				Finest
Polyhedral	1.73	—	3.21	—	6.17
Tetrahedral	6.65	—	13.17	—	26.79

Cartesian hexahedral	2.39	3.97	8.04	14.23	27.52
Body fitted hexahedral	2.4	3.97	8.04	14.23	27.52
